# Artificial Intelligence for Clinical Management of Male Infertility, a Scoping Review

**DOI:** 10.1007/s11934-024-01239-z

**Published:** 2024-11-09

**Authors:** Noopur Naik, Bradley Roth, Scott D. Lundy

**Affiliations:** 1 Cleveland Clinic Lerner College of Medicine at Case Western Reserve University, Cleveland, OH USA; 2 School of Medicine, University of California, Irvine, CA USA; 3 Department of Urology, Cleveland Clinic Foundation, Glickman Urological and Kidney Institute, Cleveland, OH 44195 USA

**Keywords:** Male Infertility, Artificial Intelligence, Machine Learning, ChatGPT

## Abstract

**Purpose of Review:**

Infertility impacts one in six couples worldwide, with male infertility contributing to approximately half of these cases. However, the causes of infertility remain incompletely understood, and current methods of clinical management are cost-restrictive, time-intensive, and have limited success. Artificial intelligence (AI) may help address some of these challenges. In this review, we synthesize recent literature in AI with implications for the clinical management of male infertility.

**Recent Findings:**

Artificial intelligence may offer opportunities for proactive, cost-effective, and efficient management of male infertility, specifically in the areas of hypogonadism, semen analysis, and interventions such as assisted reproductive technology.

**Summary:**

Patients may benefit from the integration of AI into a male infertility specialist’s clinical workflow. The ability of AI to integrate large volumes of data into predictive models could help clinicians guide conversations with patients on the value of various treatment options in infertility, but caution must be taken to ensure the quality of care being delivered remains high.

## Introduction

Over the past several decades, the prevalence of infertility has remained very high and possibly even increased, with global estimates now reaching as high as 17.5% [1]. In approximately half of these cases, male infertility is implicated [2, 3]. Various etiologies comprise the multifactorial pathophysiology of male infertility, including genetic disorders such as Klinefelter syndrome, medical conditions such as autoimmune diseases and cystic fibrosis, infections including epididymitis and sexually transmitted infections, varicocele, cancer treatments, physical issues such as testicular trauma, environmental contributors (heat exposure, air pollution), endocrinopathies, and lifestyle factors [4, 5]. Despite an increasing armament of medical and surgical treatments, over half of all cases remain idiopathic with no identifiable cause, and next-generation diagnosis and treatment paradigms will become increasingly necessary to reduce the exponential global burden of male infertility.

The current clinical management paradigm for male infertility begins with a detailed reproductive history, thorough physical exam, and laboratory semen analysis. Treatments can either be targeted to the specific underlying pathophysiology (e.g. varicocelectomy or vasectomy reversal) or generalized (e.g., medications to empirically boost sperm production, lifestyle changes to improve overall health, sperm retrieval, and/or assisted reproductive technology (ART) such as in vitro fertilization (IVF) or intracytoplasmic sperm injection (ICSI) [6]. However, current treatments are significantly cost-prohibitive for a number of patients in the United States, with variable state-mandated reproductive healthcare insurance coverage and out of pocket costs frequently rising above $20,000 [7]. Moreover, diagnostic tools such as semen analysis (SA) are imperfect measures, as about 50% of patients will have no identifiable cause and instead be given the diagnosis of idiopathic male infertility [8]. Interventions such as testicular sperm extraction (TESE) and microdissection TESE (mTESE) for azoospermic men also have limited success of 40–60% [9]. Even successful sperm retrievals still require the use of IVF/ICSI, which have their own associated variable success rates [10]. As a result of these financial, logistical, and scientific challenges, patients with male infertility experience a significant psychological burden that is often inappropriately stigmatized and minimized [11]. It is for this constellation of reasons that novel diagnostic and treatment tools to improve the efficacy of male infertility care must be developed.

**Fig. 1:**
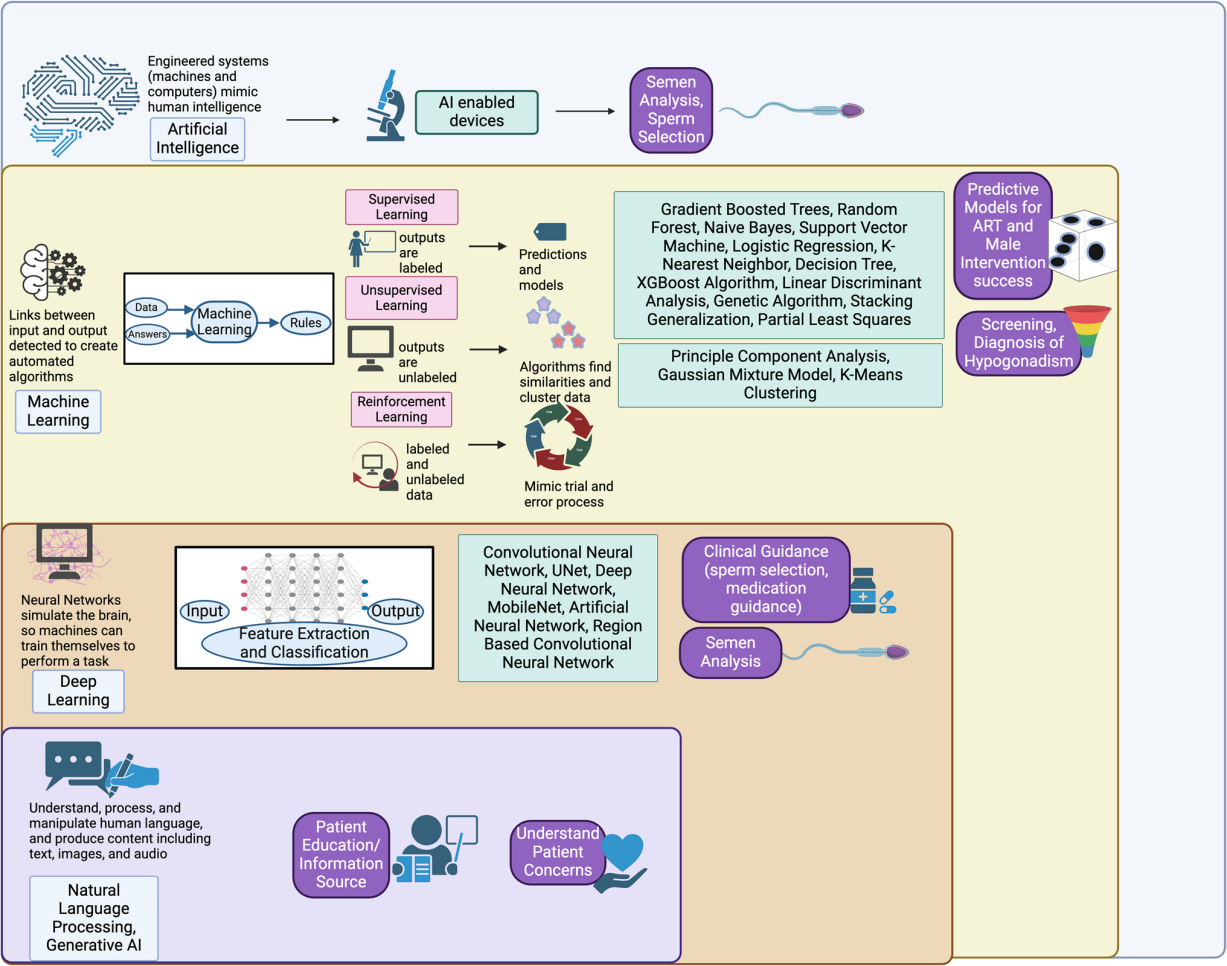
Synthesis of uses of AI in male infertility management. Machine learning is a subset of AI that involves assimilating knowledge from large amounts of data to create automated algorithms with predictive capabilities and offers the potential to identify new insights from data that may otherwise be missed. Machine learning can be supervised, in which the outputs (results) are labeled, and classification and regression algorithms are employed to address problems. Specific supervised learning models include decision trees, which are a classification technique (including random forest, gradient boosting, and decision trees including the XGBoost algorithm), AI based image recognition (which can be used in analyzing sperm), regression models, stacking generalization, support vector machines, Naive Bayes, genetic algorithm (an optimization technique inspired by natural selection), Bayesian network, and k nearest neighbors. In unsupervised machine learning, outputs are not labeled, so models can be useful for more descriptive tasks, as they can find relationships in data structures without a measured outcome. Specific unsupervised learning models include the Gaussian mixture model, K means, and principal component analysis. Finally, reinforcement learning involves a computer analyzing available data, deriving rules, and optimizing outcomes; each time the computer receives feedback about its own performance, it can improve subsequent performances via trial and error. Deep learning is a subset of supervised machine learning using an extensive neural computational network simulating the human brain and capable of discovering intricate structures in large datasets. In it, there are layers: the input layer (where information is received), the hidden layer (where processing and pattern extraction takes place), and the output layer (where final network outputs are generated). Deep learning models include artificial neural networks (a broader category of deep learning used for a variety of classification and recognition tasks), convolutional neural networks (which automatically learn spatial hierarchies of features and commonly used for image recognition; UNet is included within this), deep convolutional neural networks (which are similar to convolutional neural networks but with additional layers; including MobileNet), and region based convolutional network (which employ CNN but are suited to analyze temporal and sequential data). Finally, natural language processing leverages the ability of machines to understand, process, and manipulate human language to transcribe audio to text or to extract data from writing, while generative AI can produce content such as text, images, and audio. Examples of NLP are social media and theme analysis, and ChatGPT uses both NLP and is an example of generative AI. *Created in **BioRender.com*

The application of artificial intelligence (AI) to medical care and male infertility in particular represents an exciting new frontier for the field. In simplest terms, AI refers to the use of computers to model intelligent behavior with minimal human intervention [12, 13]. While often discussed as a single entity, AI spans several approaches including machine learning, deep learning, generative AI, and natural language processing (Fig. 1). Each of these applications has the potential to revolutionize healthcare by enabling the analysis of large datasets, the automation of manual processes, and the development of predictive modeling (Fig. 1, Table 2) [12–24]. In 2021, the FDA outlined the “Artificial Intelligence and Machine Learning Software as a Medical Device Action Plan” to regulate the implementation of AI in medical devices and digital health [12]. Currently, the FDA has approved 882 AI-enabled devices, of which 9 have pertinent uses in the field of urology [13]. Seven of these are radiology processing and analysis tools that focus on prostate cancer, while two are computer-aided semen analysis hardware. These approvals represent an increasing adoption of AI in clinical practice and demonstrate the potential to incorporate AI into the workflow of male fertility specialists.

Within male infertility, a number of recent publications have developed and tested next-generation AI technology and their predictive capability for this disease. This review aims to explore contemporary literature on the application of AI in the clinical management of male infertility with a focus on pertinent topics such as hypogonadism, semen analysis, assisted reproductive technology, male factor interventions, and AI-driven patient communication.

## Methods

A review was conducted with the goal of characterizing the value of artificial intelligence in the clinical management of male infertility. This review aimed to evaluate primary literature which used AI to provide clinically valuable insights within the subjects of hypogonadism, semen analysis, assisted reproductive technology, varicocele, and ChatGPT. The literature search covered studies available on PubMed published primarily between April 2019 and April 2024. Only primary studies were included, and papers with a basic science focus were excluded, as they were not within the scope of this review. The specific search strategy is detailed in Table 1.

**Table 1:** Methods. The following criteria were used to identify papers relevant to AI for the clinical management of male infertility. Articles were excluded for the reasons outlined in the table

Section	Search Terms	Articles Retrieved	Articles Included	Exclusion Reasons
Semen Analysis	(((artificial intelligence) OR (machine learning)) AND (semen analysis)) AND (("2019/04/30"[Date—Publication]: "2024/04/30"[Date—Publication]))	108	21	· Animal studies · Reviews · Lack of relevance to clinical management · Use of AI to evaluate a secondary or tertiary variable (other than semen)
Hypogonadism	(((artificial intelligence) OR (machine learning)) AND (hypogonadism)) AND (("2014/04/30"[Date—Completion]: "2024/04/30"[Date—Completion]))	10	7	· Lack of relevance to clinical management · Reviews
Assisted Reproduction and Interventions	(((artificial intelligence) OR (machine learning)) AND ((Testicular sperm aspiration) OR (Percutaneous Epididymal Sperm Aspiration) OR (Testicular sperm extraction) OR (varicocele) OR (Microepididymal Sperm Aspiration) OR (Microdissection TESE))) AND ((“2019/04/30”[Date – Publication]: “2023/04/30”[Date – Publication]))	21	15	· Lack of clinical relevance to male infertility management · Review
ChatGPT and Language Processing	(ChatGPT) AND ((andrology) OR (infertility)) AND ((“2019/04/30”[Date – Publication]: “2024/04/30”[Date – Publication]))	8	4	· Review · Outside scope of clinical relevance

### Semen Analysis

Semen analysis is a cornerstone of both the diagnostic process and clinical management of male infertility; it is the most frequently used diagnostic method of male infertility and is also relevant in sperm preservation, donation, and post-vasectomy screenings [25, 26]. Manual semen analysis includes an evaluation of macroscopic parameters (such as liquefaction, viscosity, ejaculate appearance, volume, and pH) as well as microscopic parameters (including calculation of sperm concentration, total sperm count, and sperm motility). This analysis is lab-based, labor intensive, and subjective; as a result, results can be inconsistent and interlaboratory variation is high, leading to potential misdiagnosis and delayed treatment [26–28].

In 1980, computer-assisted semen analysis (CASA) was introduced after image digitization made computer-based image analysis possible. CASA was anticipated to increase both the speed and objectivity of sperm concentration and motility measurement and represented one of the first iterations of AI in medicine. However, obtaining accurate and reproducible results remains a challenge due to the presence of debris, particles, and non-spermatozoa cells in samples, as well as differences in CASA instruments [29, 30]. CASA results show increased variability in evaluating sperm concentration for low (< 15 million/mL) and high concentration (> 60 million/mL) species, and morphology results also show high variability due to heterogeneity. This is a significant limitation, as oligozoospermia is defined as a sperm concentration < 15 million/mL, necessitating improvements to reduce CASA variability to manage patients struggling with infertility [30, 31]. Artificial intelligence methods have the potential to help improve the issues currently in CASA so that semen analysis is more standardized, efficient, and has less variability [26, 28, 32–51].

AI allows for enhanced analysis of motility and concentration, which are important diagnostic markers in infertility evaluation. Several AI methods, including Gaussian Mixture Model, fast deep learning, region based convolutional neural network, deep convolutional network, and convolutional neural network have been used to evaluate sperm motility [28, 32, 33, 35–39]. Machine learning based spectrophotometry has been used to evaluate sperm concentration, while a smartphone based AI algorithm evaluated concentration of total sperm, concentration of motile sperm, and motility percentage [32, 40]. These methods displayed rapid results and consistency, and may outperform state of the art methods traditionally used to assess sperm in some metrics (Table 2). Hence, AI could assist with the visual complexity of sperm selection in ART, as well as the diagnosis of male infertility. These markers are predictive of successful outcomes in assisted reproductive technology; sperm motility has played a role in IVF success, while morphology has been a more relevant parameter in ICSI [52]. Thus, improved predictive analytics of these semen parameters could better allow clinicians to counsel patients on optimal ART.

**Table 2:** An overview of studies using AI for male infertility. the following studies focused on the use of artificial intelligence in the realm of male infertility. The general category of male infertility studied, AI methods, and major results are described in the table below

Section	Author, Year	AI Methods	Results
Semen Analysis	Tsai et al., 2020 [32]	Image Recognition Algorithm on smartphone-based sperm motility test (Bemaner)	AI semen analysis results correlated with male fertility expert results. For concentration of total sperm, (r = 0.65, P < .001), for concentration of motile sperm (r = 0.84, P < .001), and for motility percentage (r = 0.90, P < .001)
	Somasundaram and Nirmala, 2021 [35]	Faster Region Convolutional Neural Network with Elliptic Scanning Algorithm (Deep Learning)	Method provided an accuracy of 97.37% in human sperm classification (normal vs abnormal)
	Agarwal, et al., 2019 [26]	Artificial Intelligence Optical Microscopic based Technology	The LensHooke X1 PRO had a high degree of correlation to manual methods in concentration, progressive motility, progressively motile sperm concentration, and seminal pH (p > 0.05 in a paired t test), but total % motility and motile sperm concentration were significantly different
	Hsu et al., 2023 [51]	Artificial Intelligence Optical Microscopic based Technology	The LensHooke assay for chromatin dispersion is faster (by 32 min) than a conventional assay. DNA fragmentation index results were highly correlated between the two methods (Spearman's rank correlation, rho = 0.8517, p < 0.0001). Integration of an auto-calculation system to diagnose sperm DNA fragmentation had high agreement with manual interpretation (rho = 0.9323, p < 0.0001), and a 21% lower coefficient of variation
	Kuroda et al., 2023 [47]	Artificial Intelligence Optical Microscopic based Technology	DNA fragmentation indices from manual vs AI techniques showed strong agreement (r = 0.97, p < 0.001)
	Hicks et al., 2019 [39]	Convolutional Neural Networks (Deep Learning)	Deep learning based semen video analysis to predict sperm motility is rapid (all methods performed within 5 min) and consistent
	Yuzkat et al., 2021 [41]	Convolutional Neural Networks	Accuracy of CNN models in automating morphological classification of sperm images was as high as 90.73%
	Marin and Chang, 2021 [34]	U-Net with transfer learning (deep learning)	U-Net with transfer learning was used for human sperm segmentation and achieved a Dice coefficient of 0.96 (sperm head), 0.94 (acrosome), and 0.95 (nucleus) against manual segmentation
	Lesani et al., 2020 [40]	Artificial Neural Network	Model accurately estimated sperm concentration based on absorption spectra with 93% prediction accuracy and 100% agreement with clinical methods
	Zhou et al., 2022 [49]	XGBoost Algorithm (machine learning)	A model predicting semen quality with modifiable lifestyle factors (smoking status, alcohol consumption, sedentary lifestyle, etc.) had an AUC between 0.648–0.697 (failed model)
	Santi et al., 2020 [48]	Multiple machine learning methods: Random forest, support vector machine, k-nearest neighbor, naive bayes, linear discriminant analysis	Machine learning algorithms found 3 hematologic variables related to semen parameters (0.69 accuracy, 0.78 sensitivity, 0.41 specificity)
	Riordon et al., 2019 [42]	Deep Convolutional Neural Network	Sperm were classified into WHO categories at high accuracy (94%), true positive rate (94.1%, positive predictive value (94.7%), and F1 score (94.1%)
	Haugen et al., 2023 [28]	Deep Convolutional Neural Network	DCNN models were able to predict spermatozoa into WHO motility categories, and had strong correlation with manual assessments for progressively motile spermatozoa (Pearson’s r = 0.88, p < 0.001) and % immotile spermatozoa (r = 0.89, p < 0.001), but only moderate correlation for rapid progressive motility ((Pearson’s r = 0.673, p < 0.001). The model predicted value was within the range of interlaboratory variation for most samples
	Mahali, et al., 2023 [46]	Deep Learning Fusion Architecture (Shifted Windows Vision Transformer combined with MobileNetV3)	This approach accurately classified sperm as normal, abnormal, and non-sperm (accuracy of the best performing model ranged from 91.7%-95.4%). The model’s accuracy also outperformed benchmark models used for the same dataset
	McCallum et al., 2019 [36]	Deep Convolutional Neural Network	Results demonstrated moderate correlation (0.43) in identifying higher DNA integrity cells relative to the median
	Valiuskaite et al., 2021 [37]	Region Based Convolutional Neural Networks	Achieved 91.77% accuracy of sperm head detection with mean absolute error of 2.92 in predicting sperm head vitality, and the Pearson correlation between and predicted sperm head vitality was 0.969
	Zhao et al., 2023 [38]	Fast Deep Learning (UNet)	Achieved high sperm head segmentation accuracy where Dice coefficients was 90%, and intersection over union reached 88.5 with average cost time of 0.023 s/image
	Javadi and Mirroshandel, 2019 [43]	Deep Neural Network	This deep learning algorithm had high accuracy in detecting morphological deformities from images, where F0.5 scores for acrosome abnormalities was 84.74%, head abnormalities was 83.86%, and vacuole abnormalities was 94.65%. Images were classified in real time, making the method rapid
	Alameri et al., 2021 [33]	Gaussian Mixture Model (machine learning)	Developed optimization protocol to accurately detect motile sperm prior to tracking. Highest average accuracy was 92.3%, sensitivity was 96.3%, and specificity was 72.4%
	Ilhan et al., 2020 [44]	Support Vector Machines, MobileNet	Support vector machines obtained sperm morphology classification accuracies of 80.5% (wavelet based features) and 83.8% (descriptor based features). MobileNet achieved 87% accuracy
	Movahed et al., 2019 [45]	Convolutional Neural Network, k-Means clustering, support vector machines, multi-channel image generation algorithm	Proposed method is able to segment all parts of sperm and achieved 0.9 dice similarity for head, 0.77 for axial filament, 0.77 for acrosome, 0.78 for nucleus, 0.75 for tail, 0.64 for mid piece
Hypogonadism	Bachelot, et al., 2024 [50]	Machine Learning Model	Unfavorable machine learning scores based on a bioclinical signature (including anthropometric, metabolic, antioxidant, micronutrient, and semen parameters) were associated with high levels of DNA fragmentation (r = -0.263, p < 0.05)
	Lu et al., 2016 [53]	Decision tree	A model based on clinical variables (such as age, triglycerides, wrist circumference) had a high sensitivity (0.861), specificity (0.842), and accuracy (0.851) in predicting late onset hypogonadism
	Kim and Moon, 2021 [54]	Genetic Algorithm (machine learning strategy)	This method improved the aging male symptoms questionnaire to identify late onset hypogonadism. Limiting to a selection of 5 items, setting a threshold of 20 points, and determining a serum testosterone level of 3.16 ng/mL allowed for a sensitivity of 0.77 and specificity of 0.19
	Novaes et al., 2021 [55]	10 machine learning classifiers	Testosterone deficiency was predicted at a threshold of 0.5 with an accuracy of 81.5 (random forest), 81.0 (extremely randomized trees), 81.4 (gradient boosting), 81.5 (support vector machine), 80.7 (k-nearest neighbors), 81 (naive bayes), 81.2 (logistic regression), 81.3 (AdaBoost), 81.1 (XGBoost), 81.6 (artificial neural network), 81.8 (weighted average ensemble classifier), 81.3 (meta learning classifier). Abdominal circumference and triglycerides had the highest feature importance
	Krenz et al., 2020 [56]	Support Vector Machines and CatBoost	Compared to clinicians, machine learning models had better sensitivity in predicting Klinefelter based on patient data pre-karyotype (100% vs 87.5%, p = 0.0455)
	Nimitha et al., 2022 [57]	Deep Convolutional Neural Network	The model had an accuracy of 98.65%, F1 score of 98.86%, and kappa coefficient of 0.9894, and took 12.5 s in detecting chromosomal abnormalities
	Catic et al., 2018 [58]	Artificial Neural Networks	Classification sensitivity of feedback neural network in classifying aneuploidies based on maternal serum screening data was 99%, and accuracy was 98.8%
Male Fertility Interventions	Bachelot et al., 2023 [67]	Random Forest	Predicted successful sperm retrieval in patients with non-obstructive azoospermia undergoing TESE (AUC = 0.90, sensitivity = 100%, specificity = 69.2%)
	Zeadna et al., 2020 [68]	Gradient Boosted Trees, Logistic Regression	GBT model predicted presence/absence of sperm in testicular biopsy in non-obstructive azoospermia patients with AUC of 0.807, sensitivity of 91%, and specificity of 51%. Multivariate logistic regression did so with AUC of 0.75, sensitivity of 97%, and specificity of 25%
	Zhang et al., 2022 [69]	Logistic Regression	Circulating microRNAs in seminal plasma were identified as predictors of sperm retrieval in microTESE (AUC = 0.927)
	Xie et al., 2020 [70]	Logistic Regression	Testis specific extracellular vesicle long noncoding RNAs in seminal plasma were used to predict presence of testicular spermatozoa in nonobstructive azoospermia patients (AUC = 0.96)
	Ory et al., 2022 [71]	Random Forest	Model predicted clinically significant upgrade in semen parameter post varicocele repair (AUC = 0.72)
	Montjean et al., 2024 [72]	Artificial Intelligence Optical Microscopic based Technology	Automated single sperm selection software had comparable outcomes with ICSI compared to embryologist selected sperm (non-significant differences between the two methods)
	Lee et al., 2022 [73]	Convolutional Neural Network based on U-Net	Rare sperm from low magnification microscopy images of microTESE samples from non obstructive azoospermia patients were detected with a positive predictive value of 84.4%, sensitivity of 86.1%, and F1 Score of 85.2%
Assisted Reproductive Technology	Chen et al., 2022 [59]	XGBoost (machine learning)	Model predicted outcomes of IVF/ICSI (cumulative live birth rates) using features such as patient age, BMI, infertility type, duration of infertility, and sex hormone levels; it had AUC of 0.901
	Khodabandelu et al., 2022 [60]	Logistic regression (LR), support vector classification, random forest, Extreme Gradient Boosting (XGBoost) and, Stacking generalization (Stack)	Brier scores using these methods to predict IUI success were 0.202 (logistic regression), 0.183 (support vector classification), 0.158 (random forest), 0.129 (XGBoost), and 0.1134 (Stack)
	Kozar et al., 2021 [61]	Artificial Neural Networks, random forest, partial least squares, support vector machines	In predicting clinical outcomes of IUI, random forest model achieved AUC of 0.66, a sensitivity of 0.432, and a specificity of 0.756. PLS achieved sensitivity of 0.459 and specificity of 0.734. Remaining models had significantly lower AUC
	Peng et al., 2023 [62]	Unsupervised K means clustering	Patients in clusters with low sperm DFI and high sperm motility and concentrations had higher odds of a live birth outcome (0.733), while those in a cluster with low sperm DFI values and low sperm motility and semen concentration levels had lower odds (0.537), and those in a cluster with high sperm DFI values and low sperm motility and semen concentration levels had even lower odds (0.394)
	Ranjbari et al., 2021 [63]	Complex Network Based Feature Engineering combined with stacked ensemble	Method predicts IUI outcome with AUC of 0.84, sensitivity of 0.79, specificity of 0.91, and accuracy of 0.85
	Serdarogullari et al., 2023 [64]	Artificial neural network	AUC to predict Day 5 Blastocyst Utilization Rate was 80.2%, and ANN identified 6 independent variables of significant value to predictions
	Tian et al., 2023 [65]	Bayesian Network	The prediction accuracy of the BN model in predicting probability of fertilization failure was 91.7%
	Naelitz et al., 2023 [66]	Logistic Regression	Testosterone: Luteinizing Hormone ratio and baseline non azoospermia are significant predictors of WHO sperm concentration category upgrades following anastrozole therapy (AUC = 0.77, sensitivity of 98%, specificity of 33%)
Language Processing	Osadchiy et al., 2020 [74]	Natural Language Processing	Analysis of 199.335 Reddit posts and 6659 tweets revealed dominant themes of patient perceptions of hypogonadism on social media to be symptoms of hypogonadism, seeing a doctor, results of laboratory tests, derogatory comments and insults, TRT medications, and cardiovascular risk
	Caglar et al., 2023 [75]	Generative Artificial Intelligence	ChatGPT answered 136 frequently asked andrology questions and 87.9% of answers were correct and adequate, 9.3% were correct but insufficient, and the remainder contained both correct and incorrect information. Reproducibility of answers was > 90%
	Chervenak et al., 2023 [76]	Generative Artificial Intelligence	ChatGPT answered CDC’s 17 infertility frequently asked questions and compared to CDC responses, produced responses of similar length, factual content, sentiment polarity, and subjectivity. 6.12% of ChatGPT statements were incorrect and only 0.68% cited a reference
	Perrot et al., 2024 [77]	Generative Artificial Intelligence	Experts had a higher mean score than all AI (significantly higher than GPT 3.5 and Bard, just slightly higher than 4). Residents had a mean score higher than Bard, but lower than both ChatGPT. ChatGPT 4 was the pest performing chatbot

AI has also demonstrated efficacy in sperm morphology analysis, which remains contentious but does offer diagnostic and therapeutic value for select patients with male infertility [78]. The application of concatenated learning, deep learning, transfer learning combined with deep learning, and convolutional neural networks [34, 41–45] have been effective in helping automate the morphological classification of sperm. Another deep-learning fusion architecture was able to distinguish sperm from impurities, addressing a current limitation of CASA [46]. Additionally, deep learning can be trained to identify spermatozoa with higher DNA integrity, while AI-based halo evaluation can provide a rapid assessment of DNA fragmentation [36, 47]. Automatic morphological classification could be used to enhance the reliability of morphological semen analysis while also saving time. These AI techniques could supplement existing semen analysis methods and allow for more accurate identification of rare clinical conditions such as globozoospermia by a broader range of andrology facilities and technologies.

Predictive models may also be able to elucidate the relationship between a multitude of diverse variables such as lifestyle factors, hematologic parameters, and semen quality. For example, a study used machine learning to examine a link between hematological and spermatogenetic cells, finding that the number of lymphocytes, mean globular volume, and erythrocyte distribution were significantly related to semen parameters [48]. Another study attempted to use XGBoost, a cutting-edge machine learning algorithm, to predict semen quality based on lifestyle factors but was unsuccessful in doing so [49]. Finally, a study was able to construct a machine learning model that assessed the relationship between semen parameters and anthropometric, metabolic, and nutritional parameters. The study found that unfavorable machine learning scores were associated with high levels of DNA fragmentation [50]. These predictive models require further optimization to be relevant for clinical use but do suggest that machine learning could be used to counsel patients on factors which may predict semen quality. Future AI models may be able to guide reproductive urologists in predicting which patients will have the most optimal semen quality that will be responsive to infertility treatments.

The LensHooke X1 PRO semen quality analyzer is an FDA approved AI optical microscope and analysis platform to assess sperm concentration, percent motility, and seminal pH with a high degree of correlation compared to manual semen evaluation (statistically insignificant difference between both methods for these parameters) [26]. LensHooke can also be integrated into diagnosis of sperm DNA fragmentation, and provide a fast, objective, and standardized method of sperm DNA fragmentation (40 min compared to 70 min conventionally, see Table 2) [51]. This system’s efficiency and ability to standardize results highlight the value of integrating an AI based system into clinical management of male infertility, as rapid test results could be immediately discussed with patients to streamline clinical workflows. By enabling more precise, efficient, and standardized evaluations, AI can significantly reduce the time to a definitive diagnosis of infertility and lead to earlier implementation of treatments. Whether the platform is fully able to overcome the challenges with prior CASA technology, however, remains to be fully understood.

In summary, the use of AI for semen analysis does indeed hold promise, but the current limitations of these technologies must be acknowledged as none have been fully evaluated for efficacy in randomized, controlled trials. Thus, their present impact is largely speculative and more robust research is required before incorporation into routine clinical practice.

## Hypogonadism

Present in up to 40% of males presenting with infertility, hypogonadism is a key comorbidity in both the development and treatment of male infertility, as testosterone is the major androgenic hormone involved in spermatogenesis[79]. Under American Urological Association (AUA) guidelines, hypogonadism is defined as a total testosterone level below 300 ng/dL made on two separate testosterone measurements combined with symptoms such as low libido, fatigue, or erectile dysfunction [80]. Etiologies of hypogonadism are broad and can be broadly described as primary (due to the issue originating in the testicles) or secondary (due to an endocrine issue in the pituitary or hypothalamus) [81]. Treatment of hypogonadism in the reproductive-aged male can be challenging, because traditional testosterone therapy inhibits endogenous testosterone and pituitary hormone production, lowers intratesticular testosterone, and frequently results in azoospermia. Instead, patients with hypogonadism who desire fertility can be prescribed alternative therapies including aromatase inhibitors, selective estrogen receptor modulators, and/or Hcg [80]. Given the prevalence of hypogonadism and the complexity of its treatment, hypogonadism has been the focus of several studies in artificial intelligence [50, 53–58].

One significant application of AI in this setting is predictive modeling to optimize screening tools for hypogonadism and testosterone deficiencies. For example, Klinefelter syndrome is the most common sex chromosome disorder in males, but historically the diagnosis was not made until adulthood due to its variable clinical presentation and low symptom presentation early in life [82, 83]. Tools to increase early diagnosis could help patients plan for fertility preservation when such treatments are most likely to be successful. AI models have been able to predict the presence of Klinefelters, and may be able to serve as a tool to inform clinicians about the likelihood of Klinefelter. For example, one neural network model was able to classify aneuploidies (including Klinefelter) based on data from a first trimester screening (including maternal serum pregnancy associated plasma protein, beta human chorionic gonadotropin, fetal nuchal translucency thickness, and presence of the nasal bone as determined by ultrasound) with a classification sensitivity as high as 99%, and an accuracy as high as 98.8% [58]. AI can also offer rapid automated approaches to karyotype analysis which reduce time burden and have high accuracy; for example, one study used deep learning can also classify chromosomal abnormalities through an automated chromosome karyotype architecture (accuracy = 98.65%, inference time = 12.5 s), offering value in terms of time burden and accuracy of diagnosis[57]. Machine learning can also be used to predict the presence of Klinefelters in azoospermic patients based on pre-karyotype parameters (such as age, height, testicular volume, semen volume, FSH, and LH) with a significantly better sensitivity rate compared to clinicians predicting Klinefelter based on the same factors (100% vs 87.5%) [56].

There are a number of questionnaires used in andrology that can be used to assess symptoms of hypogonadism in concert with laboratory evaluation, such as the Androgen Deficiency in the Aging Male (ADAM) questionnaire and the Aging Males’ Symptoms (AMS) scale [53]. Although questionnaires alone are insufficient for diagnosis, they are relevant in the diagnostic process, as they offer insight into symptom burden which must be considered alongside serologic criteria. AI can improve both the administration and interpretation of these questionnaires by identifying patterns and correlations that traditional statistics may miss; for example, one study used machine learning to identify the items on a questionnaire most significant in predicting late onset hypogonadism [53, 54].

Finally, management of male infertility can be challenging due to the large volume of variables that impact testosterone levels (such as BMI, lifestyle, health history, and time of day) [84]. AI models can predict outcomes based on these variables, which could help clinicians have conversations with patients about factors contributing to patients’ low testosterone. For example, a machine learning model identified abdominal circumference and triglycerides as significant predictors of testosterone deficiency [55]. Another study used a validated machine learning score combining anthropometric, metabolic, and nutritional parameters to predict semen parameters and distinguish men with idiopathic infertility based on a clinical signature [50]. These studies illustrate AI’s ability to integrate diverse parameters and provide a more holistic assessment of male infertility, which could both serve as a predictive tool for semen parameters and testosterone levels, and a method for clinicians to make recommendations to patients on factors which may indirectly contribute to hypogonadism induced infertility (for example, dietary changes). It is important to separate studies which may offer a new approach to making these diagnoses but ultimately will not impact patient care, versus those which may offer cost or time savings and contribute meaningfully in this regard.

## Male Factor Interventions

To address male factor infertility, current interventions include testicular sperm extraction (TESE), microscopic testicular sperm extraction (microTESE), and varicocelectomy. However, the success rate of surgical sperm retrieval is no higher than 50–60% even in ideal settings, leading to uncertainty in accurate counseling on treatment success [85, 86]. As a result, the predictive capacity of artificial intelligence is vital.

Varicocele is the most common correctable cause of male infertility, with several semen parameters being shown to improve post-varicocelectomy [71]. However, around 50% of patients show no meaningful improvement in semen parameters post-operatively due to reasons that have not yet been fully elucidated. Prior studies have applied a random forest supervised machine learning model to determine whether a patient has a low, equivocal, or high likelihood of a clinically meaningful semen improvement post varicocelectomy (defined as the ability to access a form of ART previously inaccessible). Furthermore, it identified characteristics predictive of an improvement, such as FSH levels, sperm concentration, and laterality of varicocele. The application of AI and predictive modeling could significantly benefit patients considering varicocele repair as a treatment for infertility, helping manage expectations for surgery outcomes and prevent unnecessary surgery.

AI models have shown promise in predicting the outcome of TESE. A machine learning model was able to predict TESE outcome using pre-op data (urogenital history, hormonal data, genetic data) with high AUC and sensitivity (Table 2), while another model used logistic regression to predict probability of a successful TESE [67, 68]. Other predictive models have identified microRNAs and long noncoding RNAs as biomarkers and used them to develop models which can score patients to predict sperm retrieval outcome [69, 70]. These models appear to have high accuracy (see Table 2,AUC = 0.96 for long noncoding RNAs), and once validated, could be used to guide patient selection and counseling.

## Assisted Reproductive Technologies

The use of assisted reproductive technologies (ART) is increasing at a rate of 5–10% per year [10]. Currently, 28% of ART cycles in couples are performed for a contributing male factor [87]. Hence, there is significant value in AI, which can include predictive tools to enhance understanding of the probability of success, descriptive models which could contribute to knowledge about factors influencing success, and tools to help with ART selection [59–66, 72, 73].

Thus far, XGBoost, Random Forest, K means clustering, combined complex network based feature engineering and stacked ensemble, artificial neural network, and Bayesian network model have been used to predict outcomes of IVF, ICSI, and IUI [59–65]. Many of these models performed well, and identified factors highly predictive of outcomes relevant to male infertility, such as sperm DNA fragmentation index [62] and well-established parameters such as sperm motility and concentration. Recent research also demonstrates that AI can be more practically applied in clinical management. For example, logistic regression was used to identify factors predictive of patient factors associated with improvement in semen parameters in infertile men treated with anastrozole. This type of model could be used to counsel patients on treatments that are most likely to benefit them [66]. These models could be used to identify patients who are most likely to benefit from various forms of ART, decreasing the cost and time burden that may otherwise be spent on a method which may not ultimately result in pregnancy. This is a significant consideration, as the cost per live birth of IVF can exceed $60,000 in some parts of the country [88].

It is possible that AI could be used for sperm selection to improve ICSI outcomes. One study compared AI assisted sperm selection software with embryologist selection and found similar outcomes between embryologist and AI chosen sperm, indicating that AI may be nearing human predictive ability and provides an opportunity to overcome bias and inter-examiner variability while also saving time [72]. AI is also able to automate the identification of rare sperm following microTESE, which is currently a time intensive and error prone manual process [73]. The use of AI would allow for an increased likelihood of capturing rare sperm, thereby increasing success rate and reducing time required for sperm retrieval following microTESE.

Collectively, these studies underscore the transformative potential of AI in ART, offering advanced tools for optimizing patient selection, predicting treatment outcomes, and improving procedural success rates. By integrating AI into clinical practice, reproductive specialists can improve patient expectations during shared decision making conversations with patients as well as reduce the required labor time and cost in the intervention workflow.

## ChatGPT and Language Processing

Since 2023, the number of google search results for “infertility” has increased from 1.1 billion in 2023 to 3.36 billion in 2024[76]. This illustrates the vast amount of public interest in this topic. Patients are likely to consult the internet with their questions pertaining to infertility, and may also employ the use of Chatbots, which have access to large stores of information. Several studies have assessed the performance of ChatGPT in answering frequently asked andrology questions [75–77]. These studies have found that ChatGPT provides largely accurate and reliable answers (see Table 2), with appropriate length, factual content, sentiment polarity, and subjectivity [76]. However, experts still scored higher than chatbots, and there was variability in the comparative performance of different chatbots as there was no significant difference between experts and ChatGPT 4.0 (a new and more advanced version), while experts did score significantly higher than ChatGPT 3.5 and Bard [77].

Nonetheless, the most pressing concern for the use of Chatbots in male infertility is that responses do not reliably cite sources, and there is a possibility of unpredictable fabricated information, which limits clinical use. Providers should advise patients to exercise caution when using chatbots and continue to implore seeking treatment advice from licensed reproductive specialists. Patients may also seek advice from online forums such as Reddit. To evaluate this, a study used AI to analyze themes from Reddit and Twitter, and found that common themes included hypogonadism symptoms, seeing physicians, lab test results, derogatory comments, medication, and cardiovascular risk [74]. Hence, the AI based analysis of themes on social media can help providers have a general understanding of patient concerns that patients may be otherwise apprehensive to bring up in a clinic.

## Conclusion

Integrating AI in the clinical management of male infertility presents an inevitable transformative approach to optimizing semen analysis, assisted reproductive technology, and patient communication. Algorithms utilizing machine learning have the potential to optimize sperm selection, predict treatment outcomes, and evaluate sperm DNA integrity in a more rapid manner than current manual approaches. This enables a more individualized approach to infertility which allows clinicians to have meaningful conversations with patients about the feasibility and likelihood of clinical interventions in treating infertility. Similarly, the application of AI in ART has shown potential to optimize sperm selection for IVF and IUI and predict treatment success. The use of AI requires further refinement and research to understand scalability in infertility clinics, which may be limited by funding and equipment. However, they provide valuable information which could help patients understand the likelihood of treatment success and offer insights that help optimize treatment outcomes. Nonetheless, the use of AI in medicine is still rooted in research, and not currently in guidelines for clinical management. Collaboration with healthcare professionals during AI model development and regular updates to these models in line with current research findings is crucial in ensuring that models are reliable, effective, and may be adopted into clinical workflow.

## Key References

- Joshi G, Jain A, Araveeti SR, Adhikari S, Garg H, Bhandari M. FDA-Approved Artificial Intelligence and Machine Learning (AI/ML)-Enabled Medical Devices: An Updated Landscape. Electronics. 2024 Jan;13(3):498.
  o Joshi et al. provide a comprehensive update of the current state of FDA approved AI and ML devices as of 2024. They provide a critical lens on the publicly available information from the FDA in terms of which devices are approved, their use cases, and their timeline for approval. They helpfully discuss AI approvals by specialty, which highlights the relative deficit in AI approved devices within the field of urology compared to other specialties. There are a total of 691 FDA approved AI and ML devices, of which only 11 are in the “urology/gastroenterology” category. On further examination, the devices we discussed which are actually relevant to male infertility (LensHooke) are actually grouped within the “hematology” section of the FDA’s panel. The study discusses the reliance of many of these approvals on the 510(k)-clearance pathway, and gives a helpful outline of current trends in FDA approved devices.
- Ory J, Tradewell MB, Blankstein U, Lima TF, Nackeeran S, Gonzalez DC, et al. Artificial Intelligence Based Machine Learning Models Predict Sperm Parameter Upgrading after Varicocele Repair: A Multi-Institutional Analysis. World J Mens Health. 2022 Oct;40(4):618–26.
  o Ory et al. developed an AI based ML model to predict upgrades in sperm parameters post varicocelectomy. Surgical interventions to address male infertility are variable in their success. This paper defines success as an upgrade in semen parameters (whereby a patient is able to access a method of ART previously inaccessible), and their model is able to predict clinically significant improvements in semen parameters post varicocele repair. The output of this paper will also be published as an online calculator for physician use. Hence, this represents an example where a model could provide clinically significant information to a physician in terms of likelihood of success post intervention which could then be used to guide clinical conversations and decision making.
- Perrot O, Schirmann A, Vidart A, Guillot-Tantay C, Izard V, Lebret T, et al. Chatbots vs andrologists: Testing 25 clinical cases. Fr J Urol. 2024 Jun 1;34(5):102,636.
  o Perrot et al. test the performance of andrologists compared to 2 versions of ChatGPT, and the Bard chatbot in responding to andrology clinical cases. The study found that experts had a higher mean score than all AI (significantly higher than GPT 3.5 and Bard, just slightly higher than GPT 4). Residents had mean score higher than Bard, but lower than both ChatGPT, and overall, GPT4 was the best performing chatbot. This study highlights important considerations for the implications of chatbots in andrology. It demonstrates that not all chatbots perform with similar efficacy, and thus an adoption of chatbots into clinical workflow must account for this. Additionally, as patients may start using chatbots to ask questions, clinicians should advise patients to exercise caution if and when they do use chatbots.

## Data Availability

No datasets were generated or analysed during the current study.
